# Break CDK2/Cyclin E1 Interface Allosterically with Small Peptides

**DOI:** 10.1371/journal.pone.0109154

**Published:** 2014-10-07

**Authors:** Hao Chen, Yunjie Zhao, Haotian Li, Dongyan Zhang, Yanzhao Huang, Qi Shen, Rachel Van Duyne, Fatah Kashanchi, Chen Zeng, Shiyong Liu

**Affiliations:** 1 Department of Physics, Huazhong University of Science and Technology, Wuhan, Hubei, China; 2 Department of Physics, The George Washington University, Washington, D. C., United States of America; 3 BNLMS, Center for Quantitative Biology, Peking University, Beijing, China; 4 George Mason University, National Center for Biodefense & Infectious Diseases, Manassas, Virginia, United States of America; 5 The George Washington University Medical Center, Department of Microbiology, Immunology, and Tropical Medicine, Washington, D. C., United States of America; Bioinformatics Institute, Singapore

## Abstract

Most inhibitors of Cyclin-dependent kinase 2 (CDK2) target its ATP-binding pocket. It is difficult, however, to use this pocket to design very specific inhibitors because this catalytic pocket is highly conserved in the protein family of CDKs. Here we report some short peptides targeting a noncatalytic pocket near the interface of the CDK2/Cyclin complex. Docking and molecular dynamics simulations were used to select the peptides, and detailed dynamical network analysis revealed that these peptides weaken the complex formation via allosteric interactions. Our experiments showed that upon binding to the noncatalytic pocket, these peptides break the CDK2/Cyclin complex partially and diminish its kinase activity *in vitro*. The binding affinity of these peptides measured by Surface Plasmon Resonance can reach as low as 0.5 µM.

## Introduction

Protein-protein interactions play critical roles in many biological processes, and therefore may become the targets for drug design [1]–[4]. In this approach, functional proteins [5], [6] and small inhibitors [7]–[10] are successfully designed by grafting, docking and high-throughput NMR screening.

The main strategies for designing effective peptide inhibitors fall into three categories: 1) Cutting peptide sequence [11], [12] from native protein-protein interface; 2) Phage display [13]–[16]; and 3) Computational design, including docking [17]–[20], molecular dynamics simulation [21]–[23], normal mode analysis [24], template-based searching [25] and sequence design [26]–[28]. Peptide inhibitors derived from natural protein-protein interfaces are found to disrupt protein-protein interaction [11], [12], [29], [30]. For example, Schon *et al.* [11] cut parts of P53 (sequence 15–29) and tested their binding with MDM2. They found that the peptide PMD2 (ETFSDLWKLL, K_d_ = 46 nM) bound MDM2 stronger than peptide PMD1 (SQETFSDLWKLLPEN, K_d_ = 580 nM). However, sometimes this cutting strategy does not work. For example, Gondeau *et al.* [12] found the peptide C4 derived from Cyclin A with IC_50_ = 1.8 µM does not disrupt CDK2/Cyclin A complex. Besides this “cutting” strategy, Hu *et al.* [13] found a peptide pDI (LTFEHYWAQLTS) with the ability to disrupt P53-MDM2 interaction by phage display. And then, using the same technology, Pazgier *et al.* [16] found a novel peptide PMI (TSFAEYWNLLSP, K_d_ = 3.4 nM) bound with MDM2 stronger than the wild p53 peptide (ETFSDLWKLLPE). Later, Li *et al.* [15] reported that systematic alanine scanning on PMI resulted in a mutant N8A that is the strongest binder with MDM2 (K_d_ = 490 pM). Phage display is a useful method for designing peptide inhibitor of protein-protein interaction, but it is limited to the size of the random library. It cannot cover the entire sequence space. Though the alanine scanning could make up for a number of shortcomings of the phage display technology, the optimized peptide sequence may still not be found without the help of theoretical computational method.

Structure-based computational design of inhibitor has been studied for many years. Protein-peptide docking is one such method [31], [32]. London *et al.* [19] cut the peptide from protein-protein interface in protein-protein docking benchmark 3.0 and CAPRI targets, and docked the peptide to the protein by FlexPepDock [17]. They showed that the derived peptides contributed dominantly to binding free energy, however, it is necessary to validate experimentally if such peptides actually bind their targets. In 2008, Fu *et al.* [24] successfully designed a 26-mer peptide by modeling backbone flexibility with NMA (normal mode analysis) from Bcl-X_L_/Bim-BH3 complex structure. 8 of their 17 designed peptides are validated experimentally to bind well with Bcl-X_L_. This approach relies on the knowledge of the protein-peptide structure. In most cases, peptide binding does not induce large conformational changes [33]. However, in the case of CDK2/Cyclin complex, peptide binding may induce large conformational change in its T-loop region. CDK2 inhibition and activation by phosphorylation have been studied by using 1–3 ns molecular dynamics simulations [34], which showed that its glycine-rich loop moves away from the ATP binding pocket. The T-loop is extremely flexible in the unbound state but rigid in any of the CDK2/Cyclin complexes [35]. The previous study shows that the active site cleft is blocked by the T-loop and becomes accessible to the substrate only after activation by Cyclin binding. From its inactive to active conformation, the CDK2 needs to bind Cyclin with a large conformational change in the T-loop region. Finally, its complete activation is achieved by phosphorylation at Thr160 in the T-loop. Within 5 ns MD simulations on CDK2/Cyclin A, it was observed that the T-loop with phosphorylated Thr160 stayed in its active conformation and began to reconfigure with unphosphorylated Thr160 [36].

Recently, a peptide TAALS was found experimentally to break the CDK2/Cyclin interface and inhibit HIV-1 replication [19]. Two key CDK2 residues (Y180 and K178) for the binding between TALLS and CDK2 were identified because 100% and 50% loss in binding were observed for two mutants Y180A and K178A, respectively [19]. These key residues are located at a pocket near the CDK2/Cyclin interface and the T-loop of CDK2. TAALS thus disrupts the complex formation of CDK2/Cyclin by targeting a nearby pocket instead of the interface directly. This indicated that the interface residues can be affected via allosteric interactions upon peptide binding occurred at some distance away from the interface.

In this work, we present a novel strategy to design peptide inhibitors by combining a series of computational methods and experiments, including docking simulation, MD simulation, dynamical network analysis, and SPR assay. The paper is organized as follows. We first described how the peptides were selected by docking simulations. We then identified some peptides that can bind to the nearby pockets and further weaken the CDK2/Cyclin interface using molecular dynamics simulation and dynamical network analysis. Finally, we performed *in vitro* experiments to verify our predictions.

## Results

### Peptide selection

We constructed the active (PDB ID: 1FIN) and inactive (PDB ID: 1E1X) CDK2 conformations with flexible T-loop (amino acids 150–165) by Rosetta and Morph server totaling 30 models. The peptides used in docking simulation were generated by mutating the two end residues of TAALS yielding 400 double mutants. We focus on the end residues because previous studies [19] on single mutation indicated that the middle residues are conserved. See Materials and Methods for more details. The constructed CDK2 models and peptides were used as starting structures for docking simulation. The final resulting conformations from CDK2-peptide docking simulation were clustered into 10 clusters by lowest binding free energy. One typical structure (decoy) from each cluster was kept, so the ideal number of docking structures should be 30*400*10 = 120,000. However, some cases resulted in fewer than 10 clusters. The actual number of CDK2-peptide decoys turns out to be 115,976. In order to get more accurate information, we have used three different methods to identify the peptides.

### Peptide selection according to frequency analysis

We have analyzed the structural occurrence probabilities from the top 1000 protein-peptide decoys with lowest energy calculated by AutoDock. The results show that the top 3 occurrence number of SET2_06, SET3_07, SET3_09 are 528, 110, 92, respectively. So the protein conformations SET2_06, SET3_07 and SET3_09 are favorite conformations to be used to select peptides from top peptide list. Finally, 5 peptides were selected, which are RAALF, RAALG, RAALQ, FAALA, and GAALY, respectively (see Table 1).

**Table 1:** MD simulations of CDK2-peptide docking decoys.

RANK	Protein-peptidemodels	AutoDockEnergy(Kcal/mol)	Selected	Methods	MDsimulation
49	SET2_RAALF	–12.84	RAALF	Frequency	Swam away
23	SET2_RAALG	–13.11	RAALG	Frequency	Stay
3	SET3_RAALQ	–14.67	RAALQ	Frequency	Blowing up
16	SET2_FAALA	–13.3	FAALA	Frequency	Stay
4	SET2_GAALY	–14.33	GAALY	Frequency	Stay
**RANK**	**Protein-peptide** **models**	**Pmfscore** **(Kcal/mol)**	**Selected**		
7483	SET2_KAALE	–11.34	KAALE	Pmfscore	Stay
26490	SET2_DAALT	–10.37	DAALT	Pmfscore	Stay
73048	SET1_YAALE	–10.34	YAALE	Pmfscore	Swam away
73571	SET1_YAALQ	–9.99	YAALQ	Pmfscore	Stay
40624	SET2_TAALL	–9.87	TAALL	Pmfscore	Swam away
**RANK**	**Protein-peptide** **models**	**AutoDock Energy** **(Kcal/mol)**	**Selected**		
1	SET2_RAALW	–15.89	RAALW	AutoDock Energy	Stay
3	SET3_RAALQ	–14.67	RAALQ	AutoDock Energy	Blowing up
4	SET2_GAALY	–14.33	GAALY	AutoDock Energy	Stay
5	SET2_PAALA	–13.86	PAALA	AutoDock Energy	Stay
6	SET3_RAALM	–13.82	RAALM	AutoDock Energy	Stay
**CONTROL**	**Protein-peptide** **models**	**AutoDock Energy** **(Kcal/mol)**			
	SET2_TAALS	–11.28			Stay
	SET2_LAALS	–10.98			Stay
	SET2_TAALD	–11.58			Swam away & move back

RANK: The rank of the protein-peptide model sorted by AutoDock binding energy. Methods: Frequency, Pmfscore and AutoDock (details see table 2).

SET1, SET2 and SET3 have been defined as CDK2 with different T-loop conformation (see text).

CONTROL: The previous experimental result [20] shows that TAALS and LAALS bound to unphosplorylated form of CDK2, but TAALD not.

Stay: That means that the peptide is staying in the pocket during the MD simulation.

### Peptide selection according to binding energy calculation

The binding energy describes the strength of the intermolecular interactions. The ranking results show that the peptides of RAALW, RAALQ, GAALY, PAALA, and RAALM are the top 5 peptides with lowest AutoDock binding energy.

### Peptide selection according to a knowledge-based potential

The Pmfscore [37] has been used successfully for protein-protein binding energy prediction. Therefore, we apply this knowledge-based potential to re-rank the protein-peptide docking decoy to get more candidate structures. According to this new ranking result, top 5 peptides are KAALE, DAALT, YAALE, YAALQ, and TAALL, respectively.

Considering all results of the three methods above, 13 peptides were finally selected for further MD simulations as shown in Table 2.

**Table 2:** Designed peptides based on three scoring methods.

Frequency^1^	AutoDock^2^	Pmfscore^3^
FAALA	RAALM	KAALE
RAALF	RAALQ	DAALT
RAALG	RAALW	YAALE
RAALQ	GAALY	YAALQ
GAALY	PAALA	TAALL

1 Frequency: Top 5 was selected according to the number of the peptide sequence in the top 1000 lowest energy docking decoys.

2 AutoDock: Top 5 was selected according to the calculated binding energy by AutoDock.

3 Pmfscore is a statistical potential developed by Jiang *et al.* [37]. Top 5 was selected according to the Pmfscore.

### MD simulations

There may be some conformational changes of CDK2/Cyclin complex induced by peptide binding that may render the conformations obtained from docking simulations unstable since the protein is held rigid in the simulations. In order to observe the dynamical behavior, we have done MD simulations using two different sets of Van der Waals cut-off parameters to analyze the stabilities of peptides and the correlated motions of the CDK2/Cyclin interface.

**Figure 1:**
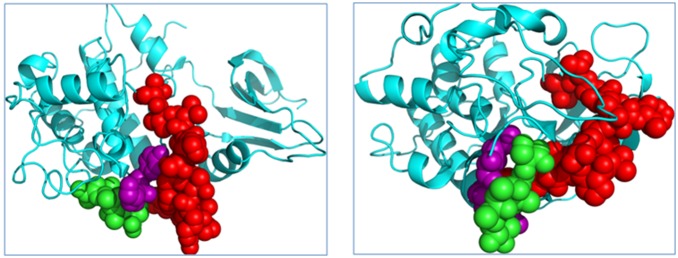
MD simulation of TAALS-CDK2 docking decoy. Left: the docked TAALS and CDK2 complex structure, as an initial structure for MD simulation; Right, after 5 ns MD simulation, the TAALS and CDK2 complex structure is shown. The green represent peptide TAALS, and the purple balls are atoms from the key residues: K178, Y180, and the red is the T-loop of CDK2. The MD simulation shows that after 5 ns, the peptide TAALS (Green) induced the conformational change of the CDK2 and moved to the gap between purple and red.

**Figure 2:**
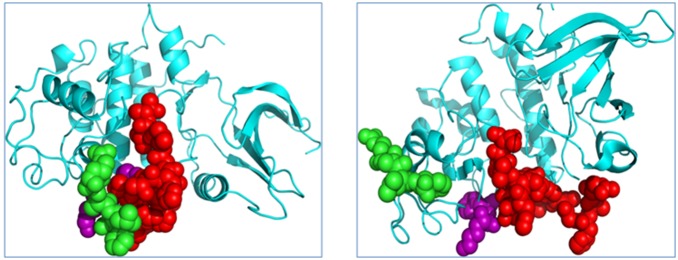
MD simulation of RAALF-CDK2 docking decoy. Left: the docked RAALF and CDK2 complex structure,as an initial structure for MD simulation; Right, after 5 ns MD simulation, the RAALF and CDK2 complex structure is shown. The green represent peptide RAALF, and the purple balls are atoms from the key residues: K178, Y180, and the red is the T-loop of CDK2. The MD simulation shows that after 5 ns, the peptide RAALF (Green) swam away from the key pocket sites of CDK2.

First, we used a sensitive cut-off 14 Å to analyze the stabilities of the 13 CDK2-peptides (shown in Table 2). As a control, we also checked the stabilities of the peptide-CDK2 complexes of TAALD, TAALS, and LAALS. The three peptides have been investigated computationally and experimentally in previous work [20], [38], [39]. TAALS and LAALS as inhibitor are found experimentally to be effective; TAALD, while having the highest predicted binding affinity, however, does not show any inhibitory effect [38]. After 5 ns MD simulations, the conformations of CDK2-peptide complex for LAALS, TAALS, DAALT, YAALQ, RAALW, RAALG, FAALA, KAALE were stable with the peptides remaining in the binding pockets. Peptide TAALD was less stable. Moreover, the peptides RAALF, YAALE, and TAALL were moving away. The MD simulations of all CDK2-peptide decoys are summarized in Table 1. For example, TAALS stayed in the binding pocket (Figure 1), however, RAALF moved away from the binding pocket (Figure 2). Finally, we selected six peptides based on these MD simulation results as summarized in Table 3.

**Table 3:** Selection based on MD simulation results.

RANK	Protein-peptidemodels	AutoDock Energy(Kcal/mol)	Selectedpeptide	Methods	MD simulation
23	SET2_RAALG	–13.11	RAALG	Frequency	Stay^1^
16	SET2_FAALA	–13.30	FAALA	Frequency	Stay between key residues and T-loop^2^
7483	SET2_KAALE	–9.35(–11.34)^3^	KAALE	Pmfscore	Stay between key residues and T-loop^2^
26490	SET2_DAALT	–7.90(–10.37)^3^	DAALT	Pmfscore	Stay^1^
73571	SET1_YAALQ	–6.05(–9.99)^3^	YAALQ	Pmfscore	Stay^1^
1	SET2_RAALW	–15.89	RAALW	AutoDock	Stay^1^

RANK: The rank of the protein-peptide model sorted by AutoDock binding energy. Methods: Frequency, Pmfscore and AutoDock (details see table 2).

SET1 and SET2 have been defined as CDK2 with different T-loop conformation (see text).

1 Stay: That means that the peptide is staying in the pocket during the MD simulation.

2 Key residue and T-loop: Key residues are that Y180, K178of CDK2.

3 The value in brackets is calculated by Pmfscore.

It is known that the ATP-binding sites of CDK2 are modified and regulated by Cyclin binding. A stable interface of CDK2/Cyclin complex is required for ATP binding and thus its enzymatic activity. In order to analyze the dynamical motions of the CDK2/Cyclin interface, we applied a method of dynamical correlation analysis to the CDK2/Cyclin interface based on the MD simulations with Van der Waals cut-off 10 Å.

**Figure 3:**
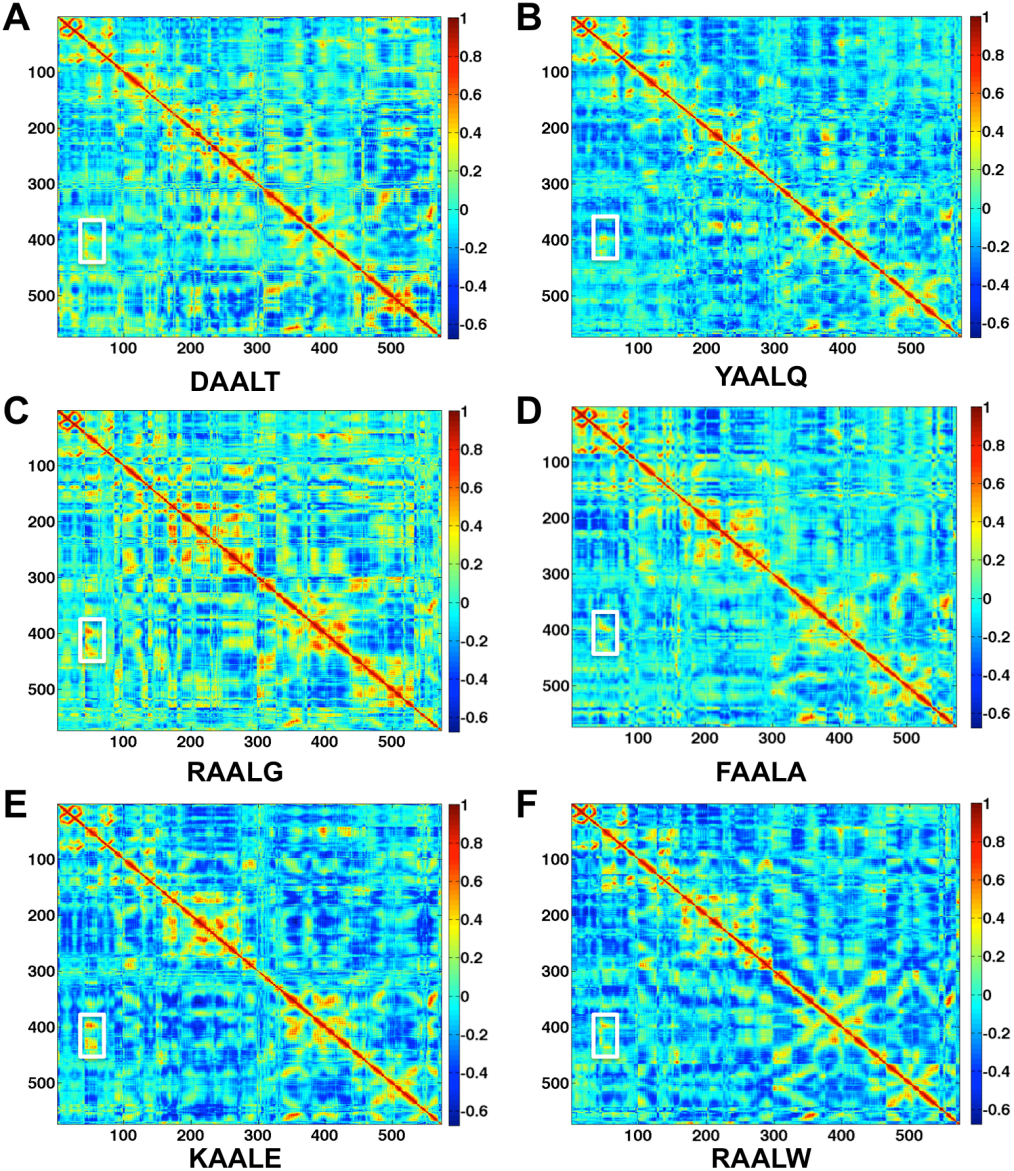
Correlation analysis of the motion during a 20-ns MD simulation of the CDK2/Cyclin/peptide complex structures. Monomers with highly (anti)correlated motion are orange or red (blue). Interface regions displaying high degree of (anti)correlation are marked in white rectangles.

If any two heavy atoms of two residues were less than 4.5 Å apart for 75% of the snapshots taken at the interval of 100 ps during 20 ns trajectories, the two residues were said to be correlated and the correlation value was computed, otherwise the correlation value was set to zero. If the residues move in the same (opposite) direction in most snapshots, the motions are defined as correlated (anti-correlated) with positive (negative) correlation values. A correlation value close to zero indicates uncorrelated motion. We focused on the residues at the CDK2/Cyclin interface. The average correlation value of the interface residues in the absence of peptide is 0.38. Figure 3 shows the correlation analysis results of the six selected peptides. The interface regions displaying high degree of correlation are marked in white rectangles. The correlation values for the cases of DAALT, YAALQ, RAALG, FAALA, KAALE, and RAALW are 0.31, 0.27, 0.44, 0.39, 0.33, 0.38, respectively. The correlation values reflect the coupled motions between CDK2 and Cyclin in the interface regions, and thus larger correlation values indicate more stable interface.

Therefore, the order of stability of the CDK2/Cyclin interface is YAALQ<DAALT<KAALE<RAALW<FAALA<RAALG. These computational results suggest that the interface regions become less stable if the peptides YAALQ and DAALT bind to CDK2. This prediction is consistent with the experimental results described in the next section. While longer MD simulations would undoubtedly provide a more pronounced correlation map, the short simulations performed here could nonetheless provide an estimate on which peptides may break up the CDK2/Cyclin interface.

### Dissociation of CDK2/Cyclin E in vitro in the presence of six designed peptides

**Figure 4:**
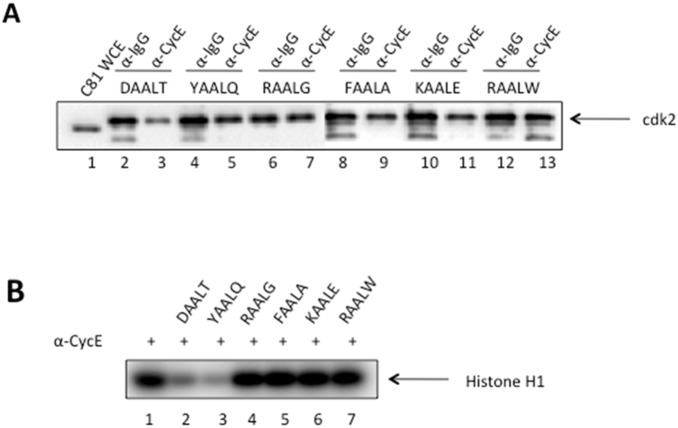
Dissociation of CDK2/Cyclin E in the presence of designed peptides. A) C81 fractionated cell extracts containing cdk2/Cyclin E complex were incubated with α-Cyclin E antibody in the presence of six designed peptides at 10 µM concentration. Following immunoprecipitation of Cyclin E, Western blot for CDK2 was shown here. α-IgG is included as a negative control. B) Immunoprecipitated Cyclin E samples in the presence of peptides were assessed for kinase activity. Histone H1 (1 µg/reaction) was added to each reaction tube along with 2 µl of (γ-32P) ATP (3000 Ci/mmol). Reactions were incubated at 37°C for 30 min and stopped by the addition Laemmli buffer. The samples were separated on a 4–20% Tris–Glycine gel. Samples were ran on a gel, dried, and exposed to a PhosphorImager cassette and analyzed using Molecular Dynamic’s ImageQuant Software.

To visualize and verify the dissociation of CDK2/Cyclin complex by each of the six designed peptides, immunoprecipitations against Cyclin and IgG, with the latter being a negative control for nonspecific background signal, were performed and followed by Western blot for CDK2 as shown in Figure 4(A). Comparing the band intensity on Lane 2 for Cyclin pulldown to that on Lane 3 for IgG pulldown, we clearly observed a weaker intensity indicating the dissociation of CDK2 from the CDK2/Cyclin complex in the presence of peptide DAALT. Upon closer inspection, the left band (lane 4) of YAALQ appears slightly wider and darker than the right band (lane 5) indicating a weak complex disassociation. However, additional evidence is needed to differentiate peptide YAALQ from other four peptides (lanes 6–13) that failed to break up the complex. This is discussed below by the kinase activity experiment.

Figure 4(B) further illustrates how the dissociation of CDK2 inhibits the kinase activity of CDK2/Cyclin complex. Here an immunoprecipitation of the CDK2/Cyclin complex was performed as previously described, followed by the kinase reaction with H1 histone being added as the substrate. The levels of phosphorylation of H1 are shown in the presence of the six designed peptides. Again, the two peptides DAALT and YAALQ exhibit a clear loss of kinase activity. To figure out how strong these two effective peptides bind to CDK2, we measured their binding affinities via Surface Plasmon Resonance as described below.

### Peptide binding measurement by a Surface Plasmon Resonance (SPR) assay

Two peptides (DAALT and YAALQ) and two positive controls (TAALS and LAALS) are tested in the same condition with CDK2 by T200 (all binding data see Table 4).

**Table 4:** SPR-derived binding affinities of CDK2 for four peptides with and without 60 µM ATP.

Peptides	K_a1_(1/Ms)	K_d1_(1/s)	K_a2_(1/Ms)	K_d2_(1/s)	K_D_(M)
TAALS	11.3±0.1	26.0±0.1 E-3	11.1±0.2 E-3	6.8±2.7 E-6	1.4±0.6 E-6
TALLS*	3.7±0.1	28.2±1.4 E-3	9.1±0.2 E-3	3.8±1.0 E-6	3.3±0.8 E-6
LAALS	498.0±8.8	11.9±0.2 E-3	5.8±2.0 E-5	2.9±0.8 E-5	8.0±2.9 E-6
LAALS*	237.4±6.9	51.8±2.3 E-3	7.9±0.5 E-3	3.1±0.3 E-3	6.1±0.6 E-5
DAALT	434.1±6.6	23.2±0.4 E-3	14.1±0.4 E-4	1.3±0.6 E-5	4.7±2.0 E-7
DAALT*	612.9±13.0	101.2±4.3 E-3	34.6±1.0 E-3	10.1±0.1 E-3	3.7±0.3 E-5
YAALQ	165.7±4.0	84.0±2.7 E-3	9.0±0.3 E-3	2.2±0.1 E-3	9.8±0.7 E-5
YAALQ*	100.9±1.5	53.9±1.4 E-3	12.3±0.3 E-3	1.6±0.1 E-3	6.1±0.3 E-5

*With 60 µM ATP.

**Figure 5:**
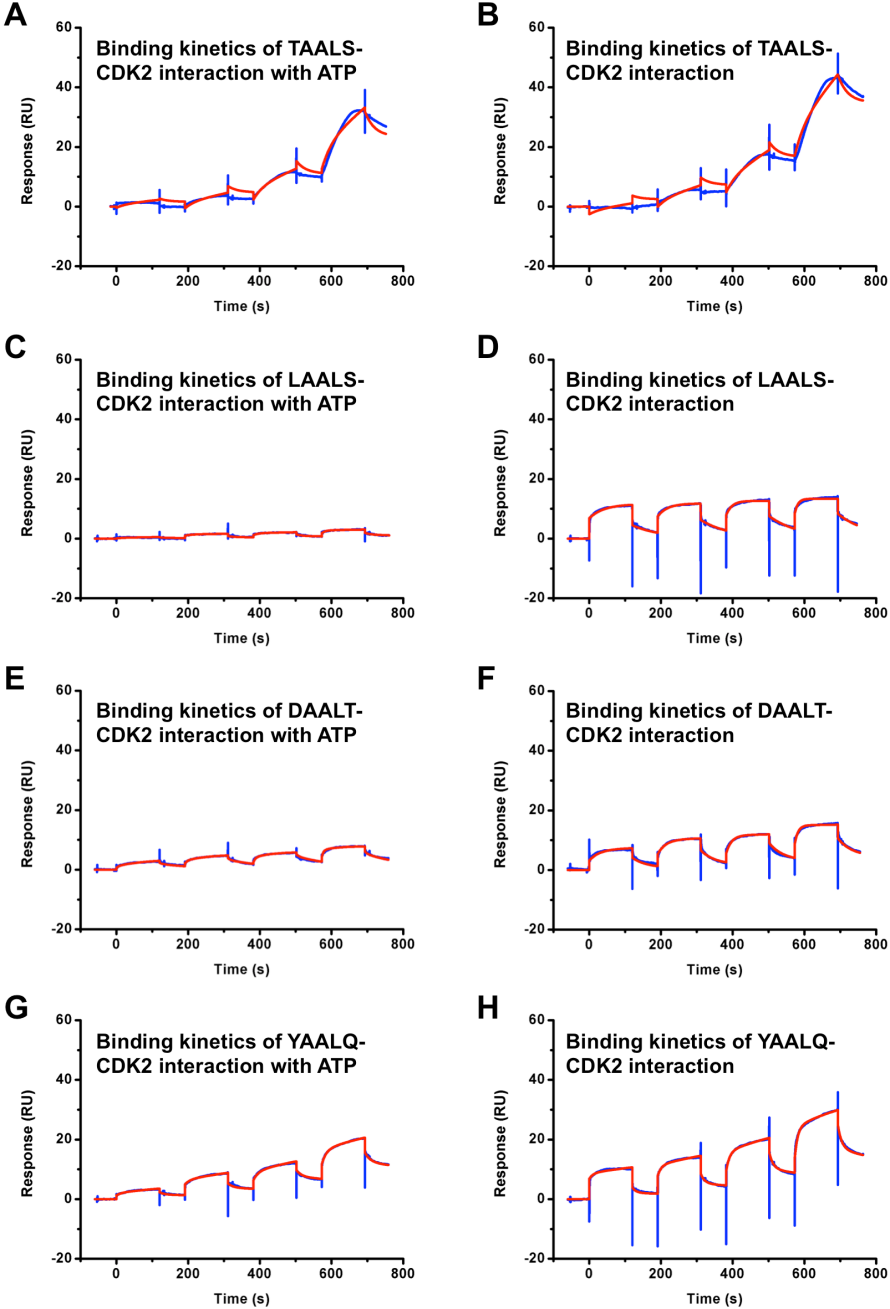
SPR binding assay results of CDK2 and peptides with and without ATP. The blue lines are experimental data, and the red lines are fitted results. The binding affinities (K_D_) between CDK2 and TAALS (A) with and (B) without 60 µM ATP are 3.3 µM, 1.4 µM, respectively. The binding affinities (K_D_) between CDK2 and LAALS (C) with and (D) without 60 µM ATP are 61 µM, 8.0 µM, respectively. The binding affinities (K_D_) between CDK2 and DAALT (E) with and (F) without 60 µM ATP are 37 µM, 0.47 µM, respectively. The binding affinities (K_D_) between CDK2 and YAALQ (G) with and (H) without 60 µM ATP are 61 µM, 98 µM, respectively.

The response curves of various analyte concentrations were globally fitted to the two-step binding model described by the following equation [40],
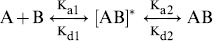
Where the equilibrium constants of each binding step are K_1_ = K_a1_/K_d1_ and K_2_ = K_a2_/K_d2_, and the overall equilibrium binding constant is calculated as K_A_ = K_1_ (1+K_2_) and K_D_ = 1/K_A_. In this model, the analyte (A) binds to the ligand (B) to form an initial complex [AB]* and then undergoes subsequent binding or conformational change to form a more stable complex AB. Data were fitted globally by using the standard two state models provided by Biacore T200 Software v2.0. The binding affinities K_D_ to CDK2 for peptides DAALT and YAALQ were measured to be 0.47 µM and 98 µM, respectively. After ATP with a concentration of 60 µM was added, the binding affinities K_D_ for peptides DAALT and YAALQ were changed to 37 µM and 61 µM, respectively. Given the large uncertainty of the fitting in SPR kinetic assays, we consider a 10-fold change in binding affinity not very significant. For the three peptides, TAALS, LAALS and YAALQ, the changes in K_D_ in the presence/absence of ATP are all within 10-fold. Thus, ATP does not have a significant effect on the binding of these peptides to CDK2. Therefore, we conclude that YAALQ does not compete directly with ATP for the ATP binding pocket. For DAALT, however, a larger-than-10-fold decrease in the presence of ATP was observed. While it is possible for DAALT to compete directly with ATP by occupying the ATP binding pocket on CDK2, it is more likely that it competes indirectly with ATP. For example, binding of ATP to CDK2 stabilizes a certain CDK2 conformation that is less favorable for DAALT binding. The detailed binding mode between DAALT and CDK2 needs to be resolved by other means beyond SPR experiments. From Figure 5, we can see that the peptide TAALS used a binding mechanism different from that of three other peptides. More precisely, upon injection of peptide, the response curve for TAALS shows a slower increase before reaching a steady value or a horizontal curve as well as a slower decrease after the peak than those for the other three peptides where a sharp jump and a dramatic drop are seen. This indicates that the mechanism of binding of TAALS represents slow binding and slow dissociation. On the contrary, LAALS displays fast binding and fast dissociation, similar to the other two peptides, DAALT and YAALQ. The SPR results confirmed a direct interaction between the peptides and CDK2. The two-state model was a better fit, suggesting that there are two different states in peptide-CDK2 binding processes. We hypothesize that the second state is the induced conformation of CDK2 by peptide binding. This is consistent with our MD simulations. The binding affinities predicted by computational docking simulations and measured by SPR between peptides and CDK2 fall into the same range (0.1 µM∼40 µM). Compared to other peptides, the association and dissociation processes of TAALS are very slow.

## Discussion

We have performed a series of computational simulations to design and select the effective peptide inhibitors against CDK2/Cyclin complex. In the structural modeling and docking selection steps, all the T-loops of the selected CDK2 were built by Rosetta loop modeling algorithm from inactive (1E1X [41]) and active (1FIN [42], Chain A) conformations. These results suggest that Rosetta loop modeling algorithm may be better for sampling flexible loop conformation than the Morph Server. In the MD simulation step, we have used two sets of Van der Waals cut-off parameters. The larger Van der Waals cut-off value considers more non-bounded interactions and is more sensitive for MD simulations. In the 5 ns MD simulations, we wanted to speed up the observation of potential instabilities of the peptides binding to CDK2/Cyclin complexes, and thus used a larger cut-off value of 14 Å. However, in the subsequent correlation analysis, we wanted to analyze the dynamical motions in detail and used the default cut-off value of 10 Å. It is generally difficult to design peptide or small molecule inhibitor to target the interface directly since typical protein-protein interface is rather diffusive. Most of the known CDK2 inhibitors target the catalytic ATP-binding pocket of CDK2 [43]. However, this pocket for the family of CDK proteins is so conserved that it is difficult to design specific CDK inhibitors for this pocket. It is thus highly desirable to discover non-ATP competitive inhibitors that function through allosteric interactions. Our previous studies [20], [38], [39] have identified such a binding pocket next to the T-loop of CDKs for designing allosteric inhibitors. A recent study by Betzi et al. [10] discovered yet another binding site near the ATP-binding pocket for designing non-ATP competitive small molecule inhibitors. Here we report a new computational methodology that combined MD simulations and novel dynamic network analysis to uncover subtle correlations not revealed by static structures in a systematic manner. This provides a means to not only identify allosteric binding sites but also understand the mechanism of allosteric interactions to design effective small molecule or peptide inhibitors. In our paper, the stability of its interface requires specific conformations of its flexible T-loop. This provide an alternative strategy to design inhibitors that disrupt the complex formation by targeting nearby pockets that could induce both the conformational changes of the T-loop and the stability of the CDK2/Cyclin interface via allosteric interactions. Equally important is the dynamical network analysis such as correlation analysis on the MD simulations because it could provide a benchmark for selecting effective peptide inhibitors.

## Materials and Methods

### Preparation of the ensemble of CDK2 and initial peptide structures

In order to model the flexibility of CDK2, T-loop (residues 150–165) conformations are reconstructed by Rosetta (version 3.1) [44] with KIC algorithm, and ten models are kept for inactive (1E1X [41]) and active (1FIN [42], Chain A) conformation, respectively. On the other hand, ten intermediate conformations of CDK2 between inactive (1E1X) and active (1FIN, Chain A) conformations are also generated by the Morph server [45] (http://molmovdb.mbb.yale.edu/). There are thirty conformations for CDK2 in total. The ten models from 1E1X by Rosetta loop prediction are named SET1; the ten models from 1FINA by Rosetta loop prediction are named SET2; and the ten models by Morph server prediction are named SET3.

Single mutation scanning experiment [20] shows that peptide (xAALx with x representing any residue) could break CDK2/Cyclin complex. So, a double mutation on position x is performed. The side-chain conformation of the double mutants are built by SCWRL4 [46]. The backbone template of the peptide used here is 1HS6A_128553_5.pdb (sequence: TAALT), which is downloaded from pepx database [47]( http://pepx.switchlab.org/ ) by query sequence pattern. AAL.

### Flexible CDK2-peptide docking based on an ensemble of CDK2

#### Docking Protocol

The peptides and CDK2 docking were performed using the Lamarckian genetic algorithm with the default parameters by AutoDock [48] (version 4.2). AutoDockTools (http://autodock.scripps.edu/) was used to prepare the ligands and the receptor. Mass-centered grid maps were generated by the AutoGrid program using the default parameters. The center of grid is set to be in the local peptide binding pocket (–12.299 28.510 35.091) in receptor (CDK2), and the number of grid points in xyz are set to be 60. The key residues (ARG150, LYS178, TYR180) in the binding pocket of CDK2, which are determined experimentally by point mutation, are set to be flexible when docking. The flexible residues of the receptor are treated in a similar way as the ligand. Hydrogen atoms were added by REDUCE (version 3.14) [49]. The results were clustered using a tolerance of 2.0 Å. Finally, 10 conformations with the lowest binding free energy were kept for further analysis.

#### MD simulation Protocol

In this study, two separate sets of MD simulations were undertaken by using the software GROMACS [50]. One is to focus on the CDK2 structures with different conformation of its T-loop; the other is for the docked CDK2-peptide structures. Each system was solvated in a cubic box with 10 Å SPC water. Then, each system was first minimized. The protein was constrained with all-bonds and the solvent molecules with counter ions were allowed to move during a 1,000-step minimization and a 100 ps long MD simulation. After relaxation, the system was simulated for 5 ns in total for all studied systems. The temperature was set at 300 K. The force field G53a6 was used in all simulations.

### Correlation Analysis

In the protein network, a node is defined as one single amino acid. If the distance of any two heavy atoms of a pair of different nodes is less than 4.5 Å for at least 75% of the snapshots, then this pair of nodes were said to form an edge [51]. The neighboring nodes in sequence are not considered to be in contact. We have done 30 ns MD simulations for each different state. The dynamical network is constructed with the final 20 ns of the 30 ns trajectories sampled every 100 ps. Then, we define the pairwise correlations (C_ij_) as 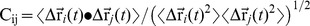, 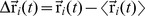, and the 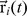 is the position of the atom corresponding to the i^th^ node. We calculated the correlations from the MD simulation trajectories using the program Carma [52].

### Cell culture

C81 is an HTLV-1-infected T-cell line that expresses Tax protein established from patients with T-cell leukemia. These cells are available through AIDS reagent catalog [53]–[55]. Cells were cultured in RPMI-1640 containing 10% fetal bovine serum, 1% penicillin/streptomycin, and 1% L-glutamine (Quality Biological) and were incubated in a 5% CO_2_ incubator at 37°C. Cells were cultured to confluency and pelleted at 4°C for 15 min at 3,000 rpm. The cell pellets were washed twice with 25 ml of phosphate-buffered saline (PBS) without Ca^2+^ and Mg^2+^ (Quality Biological) and centrifuged once more. Cell pellets were resuspended in lysis buffer (50 mM Tris–HCl, pH 7.5, 120 mM NaCl, 5 mM EDTA, 0.5% NP-40, 50 mM NaF, 0.2 mM Na_3_VO_4_, 1 mM DTT, one complete protease cocktail tablet/50 ml) and incubated on ice for 20 min, with a gentle vortexing every 5 min. Cell lysates were transferred to Eppendorf tubes and were centrifuged at 10,000 rpm for 10 min. Supernatants were transferred to a fresh tube where protein concentrations were determined using Bio-Rad protein assay (Bio- Rad, Hercules, CA).

### Peptide synthesis

All peptides used for this study were commercially synthesized (RS Sythesis, Lousiville, KY) with the following sequences:

NH2-D-A-A-L-T-OH

NH2-Y-A-A-L-Q-OH

NH2-R-A-A-L-G-OH

NH2-F-A-A-L-A-OH

NH2-K-A-A-L-E-OH

NH2-R-A-A-L-W-OH

The purity of each peptide was analyzed by HPLC to greater than 95%. Mass spectral analysis was also performed to confirm the identity of each peptide as compared to the theoretical mass (Applied Biosystems Voyager System 1042). Peptides were resuspended in dH2O to a concentration of 1 mg/ml and stored at −70C. Peptides were only thawed once prior to use for biochemical experiments.

### Size-exclusion chromatography

C81 whole cell lysate (5 mg) was fractionated on a Superose 6 HR 10/30 column (Amersham Biosciences, Piscataway, NJ) in Buffer D (20 mM HEPES (pH 7.9), 0.05 M KCl, 0.2 mM EDTA, 0.5 mM PMSF, 0.05 DTT, and 20% Glycerol). Flow-through was collected at 0.5 ml for 50 fractions. Every 10th fraction was analyzed by immunoblotting for cdk2 in order to determine the elution location of the cdk2/Cyclin E complex.

### Immunoprecipitation

Cdk2 containing chromatography fractions (28–31) were pooled together for immunoprecipitation. The pooled C81 extracts (250 µg each) were combined with 10 µM of each respective peptide. Cyclin E antibody (Santa Cruz, sc-198) was added to each reaction tube (10 µl, 2 µg), the reaction mixture was brought up to 500 µl with TNE_50_+0.1% NP-40 (100 mM Tris, pH 8.0; 50 mM NaCl; 1 mM EDTA, 0.1% Nonidet P-40) and was allowed to incubate while rotating overnight at 4°C. α-IgG was added to extract as a negative control, and an IP was performed in the absence of competing peptide, acting as a positive control. The following day, 30 µl of a 30% Protein A & G bead slurry (CalBioChem, La Jolla, CA) was added to each reaction tube and allowed to incubate while rotating for 2 h at 4°C. Samples were spun and washed 2× with TNE_300_+0.1% NP-40 (100 mM Tris, pH 8.0; 300 mM NaCl; 1 mM EDTA, 0.1% Nonidet P-40) and 1× with TNE_50_+0.1% NP-40 to remove non-specifically bound proteins. 2× Laemmli buffer was added to each sample and heated at 95°C for 3 min. Samples were loaded and run on a 4–20% Tris–Glycine SDS/PAGE gel to be used for both Western blots and kinase assays.

### Western Blot

Immunoprecipitated samples were separated on SDS/PAGE gels and were transferred to a nitrocellulose membrane via a constant current of 70 mA overnight. The membrane was blocked with a 3% BSA solution in PBS containing 0.1% Tween-20, rocking for 2 h at 4°C. A 1∶1000 dilution of α-cdk2 antibody (Santa Cruz, sc-163) was added to the blocking solution and incubated rocking overnight at 4°C. The membrane was washed with a fresh PBS+0.1% Tween-20 solution in order to wash off any residual primary antibody solution. A 1∶1000 dilution of α-rabbit secondary antibody was added to a fresh 3% BSA solution in PBS+0.1% Tween-20 and incubated with the membrane, rocking for 2 h at 4°C. The membrane was washed 2× with PBS+0.1% Tween-20 and 1× with PBS to remove any residual antibody. The membrane was exposed to chemiluminescence reagent (Pierce) in the dark for 5 min., and was developed using a BioRad Imager.

### Kinase assay

Immunoprecipitated samples were assessed for kinase activity. After the final TNE_50_+0.1% NP-40 wash, beads were washed with kinase buffer (50 mM HEPES, 10 mM MgCl2, 5 mM MnCl2, 1 mM DTT, 50 mM NaF, 0.2 mM Na_3_VO_4_ and one complete tablet of protease cocktail inhibitor/50 ml buffer) to equilibrate the reaction. Histone H1 (1 µg) was added to each reaction tube along with the γ-32P ATP (2 µl at 3000 Ci/mmol). Reactions were incubated at 37°C for 30 min and stopped by the addition of 15 µl Laemmli buffer. The samples were separated by reducing SDS-PAGE on a 4–20% Tris–Glycine gel. Gels were stained with Coomassie blue, destained, and then dried for 2 hours. Following drying, the gels were exposed to a PhosphorImager cassette and analyzed utilizing Molecular Dynamic’s ImageQuant Software.

### Peptide binding measurement by SPR

The peptides TAALS, LAALS, DAALT, and YAALQ were bought from Sangon Biotech. The purity of the four peptides were greater than 98% and they were stored at −20°C. When they were used for experiments, they are dissolved at 25°C.

Single-cycle kinetics experiments were performed with a T200 apparatus. The experiments were done on S-CM5 sensor chips coated with 6000 RU of CDK2. A flow cell left blank was used for double-referencing of the sensorgrams. Binding experiments were performed in standard PBS-P buffer with 60 µM ATP as running buffer (10 mM NaH2PO4/Na2HPO4, 150 mM NaCl, 60 µM ATP and 0.05% surfactant P20, pH 7.4) at 25°C with a flow rate of 30 µl/min. The peptide samples were prepared in the running buffer, and were injected.

The regeneration of the surface was achieved with a 30 second pulse of 20 mM NaOH. Single-cycle kinetics assay were performed using the standard SCK method implemented by the T200 Control Software. In single-cycle analysis, the analyte is injected with increasing concentrations in a single cycle. The surface is not regenerated between injections. A blank injection of buffer only was subtracted from each curve, and reference sensorgrams were subtracted from experimental sensorgrams to yield curves representing specific binding. Data were fitted globally by using the standard two-state model provided by T200 Software v2.0. The data shown are representative of at least three independent experiments.
